# Gut microbiota and immune profiling of microbiota-humanised versus wildtype mouse models of hepatointestinal schistosomiasis

**DOI:** 10.1186/s42523-024-00318-3

**Published:** 2024-06-25

**Authors:** K. A. Stark, G. Rinaldi, A. Costain, S. Clare, C. Tolley, A. Almeida, C. McCarthy, K. Harcourt, C. Brandt, T. D. Lawley, M. Berriman, A. S. MacDonald, J. E. Forde-Thomas, B. J. Hulme, K. F. Hoffmann, C. Cantacessi, A. Cortés

**Affiliations:** 1 Department of Veterinary Medicine, University of Cambridge, Cambridge, UK; 2 Department of Life Sciences, Aberystwyth University, Aberystwyth, UK; 3 Wellcome Trust Sanger Institute, Wellcome Genome Campus, Hinxton, UK; 4 Lydia Becker Institute of Immunology and Inflammation, University of Manchester, Manchester, UK; 5 Department of Medicine, Addenbrookes Hospital, University of Cambridge, Cambridge, UK; 6 Departament de Farmàcia i Tecnologia Farmacèutica i Parasitologia, Universitat de València, Valencia, Spain; 7 Present Address: Institute of Infection, Immunity and Inflammation, University of Glasgow, Glasgow, UK

**Keywords:** Host-parasite interactions, Baseline gut microbiota, *Schistosoma mansoni*, Human-microbiota associated mouse model, Bacterial 16S rRNA gene sequencing, Gut microbial diversity, Dysbiosis, Immune-modulation

## Abstract

Mounting evidence of the occurrence of direct and indirect interactions between the human blood fluke, *Schistosoma mansoni*, and the gut microbiota of rodent models raises questions on the potential role(s) of the latter in the pathophysiology of hepatointestinal schistosomiasis. However, substantial differences in both the composition and function between the gut microbiota of laboratory rodents and that of humans hinders an in-depth understanding of the significance of such interactions for human schistosomiasis. Taking advantage of the availability of a human microbiota-associated mouse model (HMA), we have previously highlighted differences in infection-associated changes in gut microbiota composition between HMA and wildtype (WT) mice. To further explore the dynamics of schistosome-microbiota relationships in HMA mice, in this study we (i) characterize qualitative and quantitative changes in gut microbiota composition of a distinct line of HMA mice (D2 HMA) infected with *S. mansoni* prior to and following the onset of parasite egg production; (ii) profile local and systemic immune responses against the parasite in HMA as well as WT mice and (iii) assess levels of faecal inflammatory markers and occult blood as indirect measures of gut tissue damage. We show that patent *S. mansoni* infection is associated with reduced bacterial alpha diversity in the gut of D2 HMA mice, alongside expansion of hydrogen sulphide-producing bacteria. Similar systemic humoral responses against *S. mansoni* in WT and D2 HMA mice, as well as levels of faecal lipocalin and markers of alternatively activated macrophages, suggest that these are independent of baseline gut microbiota composition. Qualitative comparative analyses between faecal microbial profiles of *S. mansoni*-infected WT and distinct lines of HMA mice reveal that, while infection-induced alterations of the gut microbiota composition are highly dependent on the baseline flora, bile acid composition and metabolism may represent key elements of schistosome-microbiota interactions through the gut-liver axis.

**Supplementary Information:**

The online version contains supplementary material available at 10.1186/s42523-024-00318-3.

## Introduction

Schistosomiasis, caused by blood flukes of the genus *Schistosoma*, predominantly *Schistosoma mansoni* and *S. japonicum* (causing hepatointestinal schistosomiasis) and *S. haematobium* (responsible for urogenital schistosomiasis), remains a major neglected tropical disease affecting mainly the world’s poorest communities. The infection is endemic in 78 countries across sub-Saharan Africa, South America, Middle East and South–East Asia [1–3]. Despite global efforts to eliminate this disease via mass administration of anthelmintics (i.e., praziquantel, PZQ) [4–6], re-infection rates are often staggeringly high, and emergence of drug resistance remains a tangible threat [7]. The search for alternative control strategies resulted in the identification of candidate vaccine targets (e.g., G4LZI3 protein, aspartyl aminopeptidase, microRNA-124-3p, *S. mansoni* cathepsins B1 and L3) [8–12] some of which are currently being tested in clinical trials (i.e., Sm-p80, *Schistosoma* *mansoni* tetraspanin-2 and Sm14) [13–15]. While promising, no vaccine is currently available for large-scale use, and thus new and sustainable strategies are urgently needed to control the disease and limit schistosomiasis-associated immunopathology [16].

The pathogenesis of schistosomiasis is primarily driven by the immune response mounted by the mammalian host against parasite eggs during the patent phase of infection [17, 18]. In particular, the onset of hepatointestinal schistosomiasis is associated with the formation of fibrous granulomas around worm eggs that are either swept to the liver via the portal circulation or, while undergoing migration to reach the intestinal lumen, become trapped in the gut wall. The development of *Schistosoma* egg-induced granulomas is the result of finely regulated crosstalk between egg-secreted antigens and host immunity [19]; nevertheless, increasing evidence points toward a likely role of the host gut microbiota in this crosstalk [20, 21]. First, Holzscheiter et al. [21], showed that the administration of broad-spectrum antibiotics and antimycotics to mice, followed by experimental *S. mansoni* infection, resulted in substantially decreased intestinal inflammation and granuloma development. In addition, lymphocyte preparations from mesenteric lymph nodes collected from antibiotic-treated, microbiota-depleted mice and cultured with *S. mansoni* soluble egg antigen (SEA) resulted in reduced production of IFN-γ and IL-10, thus indicating that gut resident bacteria may influence aspects of anti-schistosome immunity [21]. Building on these findings and taking advantage of the availability of high-throughput amplicon sequencing technologies for the characterisation of complex bacterial communities, we conducted an initial study to unveil the impact of *S. mansoni* infection on the gut microbial profiles of mice prior to and following the onset of egg laying [22]. Changes in the composition of the rodent gut microbiota were observed during the pre-patent period (including expanded populations of Lactobacillaceae and of the mucin degrader *Akkermansia muciniphila*); however, these were most evident after the onset of patent infection, and included features of gut microbial dysbiosis such as reduced alpha diversity and expansion of bacterial taxa with putative pro-inflammatory functions [22]. These observational studies have shed significant light on the potential contribution of the host gut microbiota to the pathophysiology of granulomatous disease caused by *S. mansoni*. Nevertheless, further mechanistic and translational investigations to unveil the roles of the host gut microbiota in disease and develop microbiome-targeting intervention strategies, respectively, are needed. The first step in this direction must take into account the considerable differences in composition and function between the gut microbiota of laboratory rodents and that of humans.

We have recently compared the gut microbial profiles of wild-type (WT) vs. human microbiota-associated (HMA) mice infected with *S. mansoni* [23, 24]. Despite substantial differences in gut microbiome composition between WT and HMA mouse lines, the relative abundances of selected *Bacteroides* (i.e., *B. sartorii* in HMA, and *B. acidifaciens* and *B. caecimuris* in WT) and *Parabacteroides* (i.e., *P. goldsteinii* in HMA and *P. distasonis* in WT), including species with pro- and anti-inflammatory functions [25], were positively correlated with *S. mansoni* infection in both groups of rodents. Nevertheless, the reproducibility of these findings must first be assessed in other HMA lines generated using faecal transplants from other human donors. Here, we characterise the changes in gut microbiota composition of a distinct line of HMA mice infected with *S. mansoni*. Furthermore, we analyse the serum antibody profiles of HMA vs. WT mice over the course of *S. mansoni* infection and characterise the levels of faecal inflammatory markers in the caecal content of these hosts. Our results support previous observations indicating that infection-associated alterations of gut microbiota composition are mostly linked to the migration of parasite eggs, albeit strongly dependent on the host baseline microbiota. However, comparison across mouse lines led to the identification of consistent alterations in gut microbiota composition that suggest that gut microbial communities may influence disease progression by affecting the gut-liver axis.

## Materials and methods

### Ethics statement

The complete life cycle of *S. mansoni* (NMRI strain) was maintained at the Wellcome Sanger Institute (WSI) and Aberystywth University (AU) by breeding and experimentally infecting susceptible *Biomphalaria glabrata* snails (NMRI strain) and mice (outbred TO line). All mouse infections and regulated procedures described in this study were presented to and approved by the Animal Welfare and Ethical Review Body (AWERB) of the WSI or AU. All experiments were conducted under Home Office Project Licenses (Procedure Project License–PPL) No. P77E8A062 held by GR, No. P6D3B94CC held by TDL and No P3BC46FD held by KFH. The AWERB is constituted as required by the UK Animals (Scientific Procedures) Act 1986 Amendment Regulations 2012.

### Generation of HMA mice and *S. mansoni* experimental infections

HMA mice were generated at the WSI as previously described [23]. Briefly, freshly collected faeces from a healthy human donor, hereafter referred to as ‘donor 2 HMA’ (= D2 HMA), were processed within 1 h from delivery to the laboratory. The sample was homogenized at 100 mg/ml in 1 × D-PBS (Dulbecco’s phosphate-buffered saline) in an anaerobic cabinet (80% CO_2_, 10% H_2_, 10% N_2_). Male and female C57BL/6 germ-free mice, bred at the WSI, were inoculated by oral gavage with 200 µl of D2 homogenate once a week, for three weeks. Thereafter, animals were removed from the isolator in sealed ISOcages and maintained on a positive pressure ISOrack (Tecniplast). HMA mice used in this study belonged to the 4th generation of breeding animals. Experimental percutaneous infections with *S. mansoni* were conducted on female D2 HMA mice, and sex- and age-matched WT, using 80 mixed-sex cercariae per mouse, as previously described [26]. Infected animals were maintained for either 28 or 50 days post-infection (= dpi; i.e., prior to and following the onset of parasite egg laying, respectively; hereafter referred to as ‘*Sm*+ _d28’ and ‘*Sm*+ _d50’); matched uninfected animals (*Sm−*) were included as controls (Supplementary Material 1).

### Parasitological analyses

*S. mansoni* adult worms were recovered from infected mice by portal perfusion [26], and livers and small intestines were removed for parasite egg counts. Control mice were perfused under the same conditions as infected animals. Infection burdens in *Sm*+_d28 and *Sm*+_d50 D2 HMA and matched WT mice were assessed based on worm numbers in individual perfusates and egg counts from liver and small intestinal tissues [26]. In brief, intestinal and right-lobe liver sections were weighed and digested overnight (~ 17 h) in 5 ml of freshly prepared 4% KOH in 1 × PBS, at 37 °C under gentle agitation. Eggs were counted in 10 aliquots of 10 μl each, and the number of eggs per gram (EPG) of intestine or liver calculated by extrapolating the mean number of eggs per aliquot to the total volume and dividing by tissue weight. The number of eggs produced by individual female worms (i.e., female fecundity), was calculated by dividing the EPG of liver and intestine by the total number of female worms recovered from each mouse. Differences in worm burdens, as well as EPG of intestine or liver, and female fecundity between *Sm*+_d28 and *Sm*+_d50 D2 HMA and WT mice, respectively, were evaluated by Mann–Whitney test, following the application of the ROUT method for outlier identification (GraphPad Prism) and the Kolmogorov–Smirnov normality test for assessment of normal distribution of worm and egg count datasets. For worm burdens, an outlier value was identified in *Sm*+_d50 D2 HMA (total worm count = 57) that was thus removed from subsequent comparative analyses with *Sm*+_d50 WT.

### Serological screening

Blood samples (50 µl per mouse) were collected from *Sm*+*/Sm−*_d28 and _d50 D2 HMA and matched WT animals by puncture of the tail vein and allowed to clot at RT for 30 min prior to centrifugation at 1000–2000 g for 10 min at 4 °C. The sera were stored at − 80 °C until further use. Specific antibody responses against *S. mansoni* SEA, soluble worm antigen preparation (SWAP) and gut bacterial antigens were assessed by indirect ELISA (Supplementary Material 2) [27]. SEA was generated at AU by isolating *S. mansoni* eggs from livers of infected mice as previously described [28] and disrupting them in PBS using a dounce homogeniser (100–200 strokes). The resulting homogenate was cleared by centrifuging at 3,600 RPM for 15 min at 4 °C, and stored at − 80 °C until further use. SWAP was generated at AU from *S*. *mansoni* adult male worms collected by portal perfusion as previously described [29], with the minor modification of using PBS as the homogenisation buffer. The resulting homogenate was stored at − 80 °C until further use. A crude bacterial antigen preparation (BAP) was obtained as described [30]; briefly, caecal content from non-experimental naïve mice were homogenised and centrifuged at 1000 g to remove large aggregates. The resulting supernatant was washed twice with PBS at 8000 g for 1 min, re-suspended in sterile 1 × PBS and sonicated on ice. After centrifugation at 20,000 g for 10 min, the protein content of all three supernatants (SEA, SWAP and BAP) was measured using the Bradford protein assay (Sigma).

Ninety-six well plates were coated overnight at 4 °C with 25 μg/ml SEA, 25 μg/ml SWAP, or 5 μg/ml BAP, and then blocked with 1% bovine serum albumin (BSA) in PBS for 90 min at RT. After blocking and between each incubation step, plates were washed 3 times with PBS containing 0.05% Tween-20 (Sigma). 50 μl of diluted serum (1/50 in PBS) were added to each well and incubated for 2 h at RT. Alkaline-phosphatase-conjugated goat anti-mouse secondary antibodies (Southern Biotech) were used to detect *Schistosoma*-specific IgG1, IgG2c, and IgG3, as well as total IgG against SEA, SWAP, and BAP. Each secondary antibody was added to plates at appropriate dilutions (Supplementary Material 2) and incubated for 1 h at RT; bound antibodies were then detected by adding liquid p-nitrophenyl phosphate substrate (Southern Biotech) and absorbance read at 405 nm using an Infinite® 200 PRO plate reader (Tecan). In addition, total serum IgE levels were measured using paired capture and detection antibodies (BD Biosciences) (Supplementary Material 2), with quantity assessed by standard curve. Levels of anti-SEA and -SWAP antibodies were compared using one-way ANOVA with Brown-Forsythe test for testing the equality of group variances, while levels of anti-BAP antibodies were compared with unpaired t-test.

### Faecal sample collection, and analysis of cytokines, inflammatory markers and occult blood

Faecal pellets were removed directly from the colons of *Sm*+*/Sm−*_d28 and _d50 D2 HMA and matched WT animals, transferred to sterile tubes and snap frozen on dry ice prior to storing at − 80 °C and processing within one month from collection (see Supplementary Material 1 for details on the number of samples processed for each assay and corresponding metadata). Faecal pellets from our previous study examining the effect of experimental *S. mansoni* infection on the gut microbiota of a distinct line of HMA mice (i.e., D7 HMA), as well as corresponding WT [23], were also obtained for comparative analyses of faecal cytokines and inflammatory markers (Supplementary Material 1). In particular, levels of faecal lipocalin-2, Ym1, RELMα, IL-33 and IL-17 in each *Sm*+_d28 and _d50 D2 HMA, D7 HMA and corresponding WT mice, as well as uninfected controls, were analysed by paired capture ELISA according to manufacturers’ instructions (Supplementary Material 2). Prior to immunoassays, faecal pellets were reconstituted in PBS containing 0.1% Tween-20 (100 mg/ml), centrifuged at 500 g and supernatants collected for analysis. Differences between groups were assessed by one-way ANOVA with Tukey post hoc test.

The presence of occult blood in faeces of *Sm*+*/Sm−*_d28 and _d50 D2 HMA and corresponding WT mice was determined by HEMDETECT® occult blood detection kit (Dipro) according to manufacturer’s instructions.

### Faecal DNA isolation, high-throughput bacterial 16S rRNA amplicon sequencing, and bioinformatics and statistical analyses

Total DNA was isolated from colonic luminal contents collected from *Sm*− and *Sm*+_d28 and _d50 D2 HMA (Supplementary Material 1), as well as no-DNA template negative controls [22] using the PowerSoil DNA Isolation Pro Kit (Qiagen) according to manufacturers’ instructions. High-throughput sequencing of the V3-V4 region of the bacterial 16S rRNA gene was performed by Novogene Europe according to established protocols (https://www.novogene.com/eu-en/services/research-services/metagenome-sequencing/16s-18s-its-amplicon-metagenomic-sequencing/#overview). Raw 16S rRNA amplicon sequencing data are available from the European Nucleotide Archive (ENA) database under project number PRJEB71733. ASV and taxonomy tables, provided by Novogene and generated using DADA2 (implemented in QIIME2 v. 2022.2) and the SILVA 138 bacterial SSU rRNA database, were formatted according to the requirements of the online software MicrobiomeAnalyst 2.0 (https://www.microbiomeanalyst.ca/MicrobiomeAnalyst/ModuleView.xhtml) and imported into the latter for subsequent analyses. Briefly, the relative abundances of individual microbial taxa in faeces from *Sm*+_d28 and _d50, and *Sm−* D2 HMA were calculated by total sum normalisation (TSS). For comparative analyses of alpha and beta diversity, as well as of bacterial taxa abundances between experimental groups, cumulative-sum scaling (CSS) was applied to the ASV table. Faecal microbial alpha diversity (Shannon index) and richness were calculated for each sample and differences between groups evaluated by ANOVA. Principal Coordinates Analysis (PCoA) based on Bray–Curtis dissimilarities between samples was performed, while differences in beta diversity were assessed using Analysis of Similarity (ANOSIM) [31]. Differences in the relative abundances of individual bacterial taxa were assessed by the Linear discriminant analysis Effect Size (LEfSe) workflow (LDA score ≥ 2) [32], as well as by Wilcoxon rank-sum and Kruskal–Wallis tests for two- and three-group comparisons, respectively. *P*-values were corrected for multiple testing using the False Discovery Rate (FDR) method and the *q*-value cut-off set at 0.05.

### Qualitative comparative analyses of alterations in gut microbiota composition associated with acute patent infection by *S. mansoni*

Significant changes in gut microbiota composition between *Sm*+_d50 and *Sm−* D2 HMA mice identified in the present study were compared to those reported in a previous study conducted in distinct mouse lines (i.e., WT and D7 HMA [23]) (Supplementary Material 1) to identify changes in the relative abundances of bacterial taxa consistently linked to acute patent infection by *S. mansoni*, irrespective of host-, parasite- and/or environment-associated variables (e.g., genetic background, infection dose, and diet). Following the criteria described in Cortés et al. [23], differentially abundant taxa displaying *p*-value < 0.05 by Wilcoxon rank-sum test and/or LEfSe (LDA score ≥ 2) between *Sm−* and *Sm*+_d50 D2 HMA mice were considered as statistically significant.

## Results and discussion

### Higher infection burdens and indicators of gut barrier dysfunction in D2 HMA mice compared to WT

**Fig. 1:**
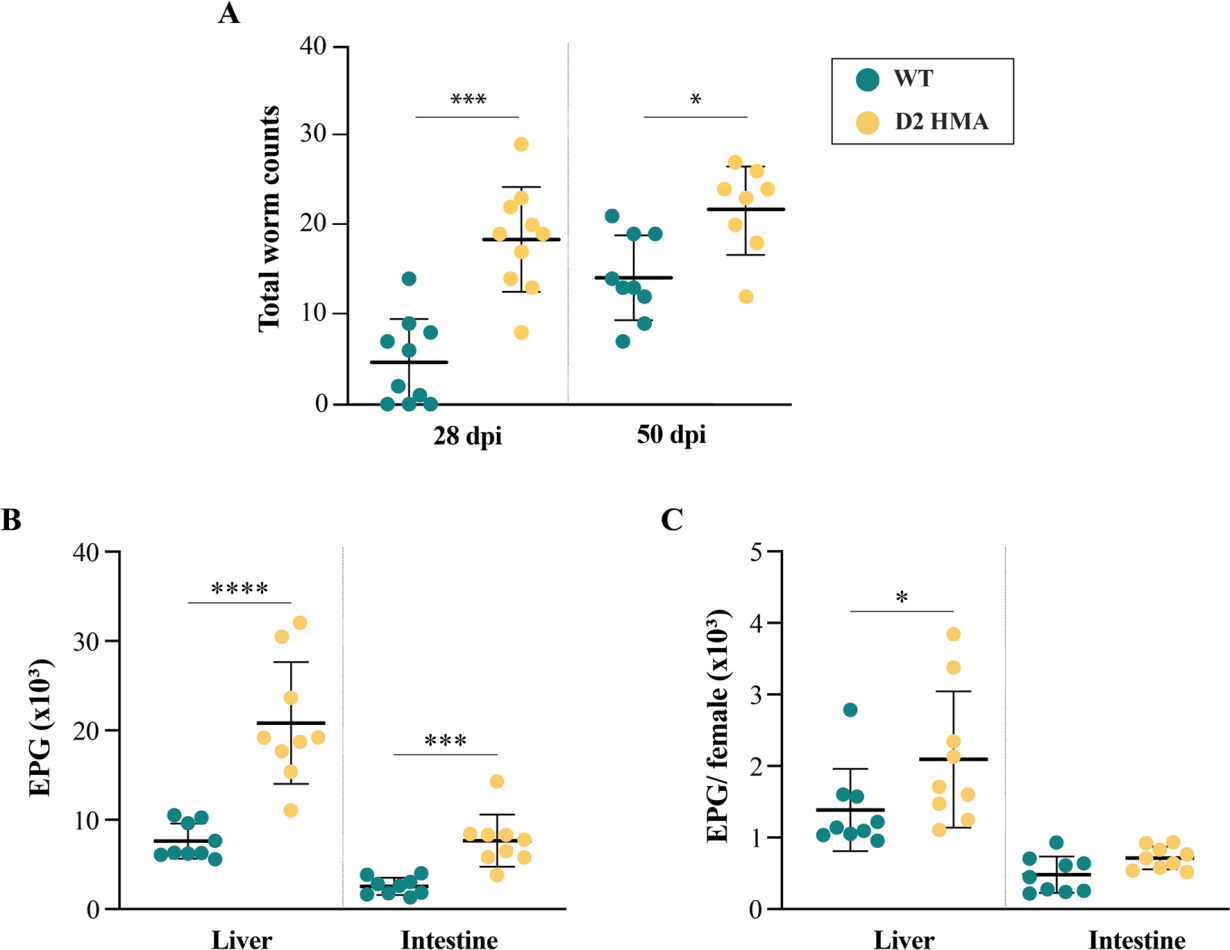
Host baseline gut microbiota composition affects susceptibility to *Schistosoma mansoni* infection. **A** Mean number (± standard error) of adult male and female *S. mansoni* recovered from wildtype (WT) and D2 human-microbiota-associated (HMA) mice at 28 and 50 days post-infection (dpi). **B** Eggs per gram (EPG) of liver and small intestine, and **C** ratio of EPG/number of female worms collected from the same animals at 50 dpi. *N* = 9–10 mice per group (i.e., mouse line and dpi). Horizontal lines indicate statistically significant differences between mouse lines as assessed by Mann–Whitney test: *p < 0.05; ***p < 0.001; ****p < 0.0001

All D2 HMA (*n* = 20) and WT mice (*n* = 20) exposed to *S. mansoni* cercariae were successfully infected, as indicated by the presence of mixed-sex adult worms in the portal system, and parasite eggs in liver and small intestine, respectively (Fig. 1). Significantly higher worm burdens at both 28 and 50 dpi, and EPG of liver and intestine at 50 dpi (i.e., patent phase), were observed in D2 HMA compared to WT (Fig. 1A and B). Moreover, a higher EPG of liver/number of female worms was observed in D2 HMA compared to WT that may suggest increased female fecundity in worms recovered from the former compared to the latter mouse line (Fig. 1C). These findings are partly consistent with data from our previous study in which higher *S. mansoni* worm and egg burdens, but not female fecundity, were recorded in a distinct line of HMA mice (i.e., D7 HMA) [23].

**Fig. 2:**
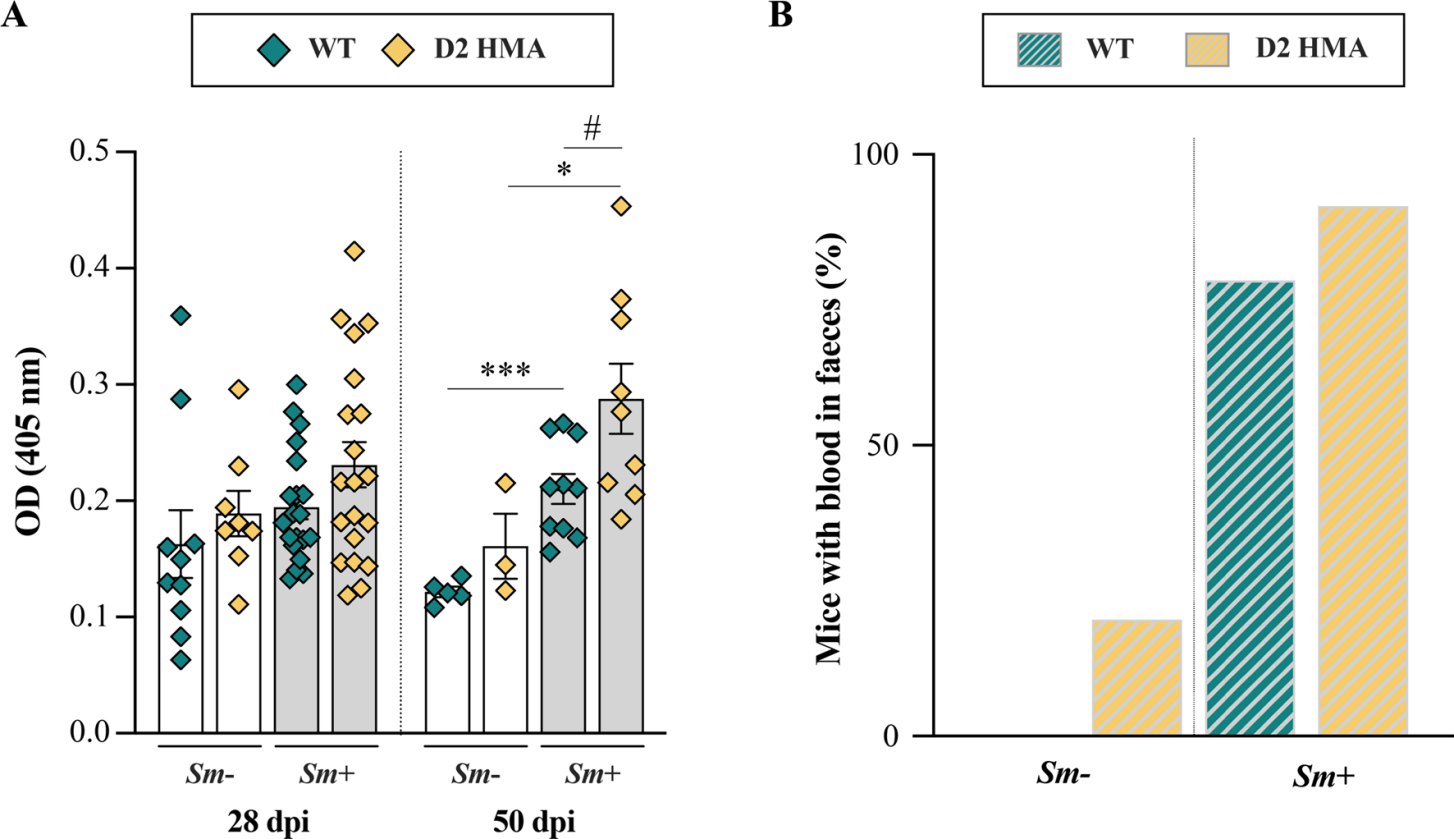
Indicators of intestinal barrier disruption in *Schistosoma mansoni*-infected wildtype (WT) and D2 human-microbiota-associated (HMA) mice. **A** Levels of total IgG against gut commensal bacteria in the serum of infected (*Sm*+) and uninfected (*Sm−*) WT and D2 HMA mice at 28 and 50 days post-infection (dpi). OD = optical density. *N* ranged between 8–10 *Sm−*, and 19–20 *Sm*+ for each D2 HMA and WT at 28 dpi, and between 3–5 *Sm−* and 9–10 *Sm*+ for each D2 HMA and WT at 50 dpi (see Supplementary Material 1). Horizontal bars and hash symbols indicate significant differences between *Sm*+ and *Sm−* animals of the same line, and between *Sm*+ animals of both mouse lines, respectively: */^#^*p* < 0.01, ***p < 0.001, determined by unpaired *t*-test. **B** Percentage of *Sm−* and *Sm*+ mice with detectable faecal occult blood at 50 dpi (*n* = 5 for *Sm−* and = 10 for *Sm*+ of each D2 HMA and WT)

The egg of *S. mansoni* secretes molecules that induce immune cell recruitment, inflammation and fibrosis that ultimately lead to granuloma formation [19]. The granuloma is exploited by the egg to migrate and traverse the intestinal wall to reach the intestinal lumen, thus causing local tissue injury [17, 18]. Therefore, we next investigated the presence of occult blood in faeces and serum antibody responses against gut commensal antigens as indirect measures of gut barrier damage in both D2 HMA and WT mice. No significant differences in total IgG levels against bacterial antigens were detected between *Sm*+ and *Sm−* of either D2 HMA and WT at 28 dpi (i.e., before the onset of egg laying) (Fig. 2A). Nevertheless, at 50 dpi, serum reactivity to bacterial antigens was significantly elevated in *Sm*+ of both D2 HMA and WT compared to *Sm−*, and accompanied by the detection of faecal occult blood in *Sm*+ samples of both mouse lines (Fig. 2B). Of note, significantly higher titres of anti-commensal IgG were detected in *Sm*+ D2 HMA compared to *Sm*+ WT at 50 dpi (Fig. 2A), a finding that may be related to the significantly higher egg burdens in the former group of animals, and that might have led to further disruption of gut barrier integrity [33].

### D2 HMA and WT mice mount similar serum antibody responses against *S. mansoni*

**Fig. 3:**
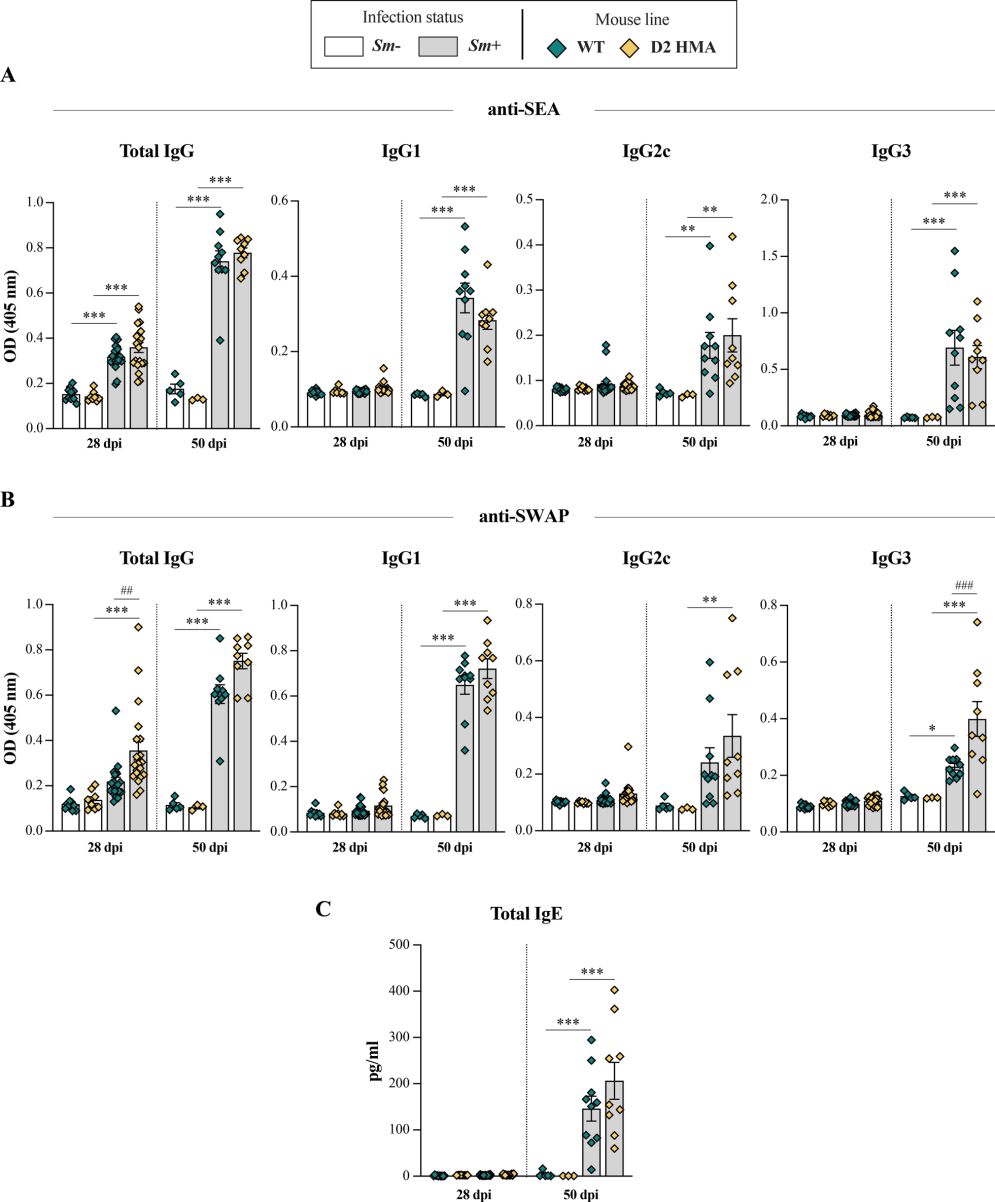
Host baseline gut microbiota composition does not affect anti-*Schistosoma mansoni* serum antibody responses. Serum levels of total IgG, IgG1, IgG2c and IgG3 specific to **A** *S. mansoni* soluble egg antigen (SEA) and **B** soluble worm antigen preparation (SWAP) in wildtype (WT) and D2 human-microbiota-associated (HMA) mice at 28 and 50 days post-infection (dpi). **C** Levels of total IgE in sera from the same animals. OD = optical density. *N* ranged between 8–10 *Sm−* and 19–20 *Sm* + for each D2 HMA and WT at 28 dpi, and between 3–5 *Sm−* and 9–10 *Sm*+ for each D2 HMA and WT at 50 dpi (see Supplementary Material 1). Asterisks indicate significant differences (as determined by ANOVA) between *Sm*+ and *Sm−* of the same line, and hash symbols between *Sm*+ of both mouse lines: *p < 0.01, **/^##^*p* < 0.001, ***/^###^*p* < 0.001

Given the striking differences in infection burdens between D2 HMA and WT mice (Figure 1), we sought to investigate specific anti-*Schistosoma* antibody responses in each group of mice. To this end, titres of total IgG, IgG1, IgG2c, and IgG3 against SWAP and SEA, as well as total IgE, were measured in sera of *Sm*+/*Sm−* D2 HMA and WT mice at 28 and 50 dpi. In mice experimentally infected with *S. mansoni*, seroconversion usually occurs at ~ 3 weeks post-infection [34]. Accordingly, significantly increased total IgG against parasite antigens was recorded in both *Sm*+_d28 D2 HMA and WT compared with *Sm−*, albeit no significant differences in titres of any IgG (sub)class, nor total serum IgE, were detected at this time point (Fig. 3). The detection of anti-SEA IgG during the pre-patent phase of infection may be linked to cross-reactivity between anti-SWAP antibodies and egg-derived molecules, or the same antigens being expressed in both developmental stages [35]. At 50 dpi, total IgG, IgG1, and IgG3 against both SWAP and SEA, as well as total serum IgE, were significantly elevated in *Sm*+ of both mouse lines (Fig. 3). Similarly, anti-SEA IgG2c were significantly elevated in *Sm*+ irrespective of mouse line, whereas IgG2c responses against SWAP were only significant in *Sm*+_d50 D2 HMA compared to their *Sm−* counterparts (Fig. 3). Titres of anti-SWAP IgG3 were higher in *Sm*+ D2 HMA compared to *Sm*+ WT at 50 dpi, similarly to anti-SWAP total IgG at 28 dpi (Fig. 3).

Comparable antibody kynetics in *Sm*+ D2 HMA and WT mice suggest that systemic humoral responses against *S. mansoni* are independent of baseline gut microbiota composition, thus supporting our previous observations [23] and indicating that the higher worm burdens observed in *Sm*+ D2 HMA are unlikely to be attributable to deficient or altered antibody responses in animals harboring a human-derived gut microbiota. Indeed, our results are consistent with early descriptions of murine antibody responses against *S. mansoni*, with serum antibody levels, and those of IgG1 in particular, increasing mainly after 40 dpi [36]. IgG1 was also identified as the predominant IgG sub-class released by acute-phase granulomas (i.e., 8 weeks post-infection) isolated from the liver of experimentally infected mice and cultured in vitro [37]*.* Moreover, increased total IgE in sera of *S. mansoni-*infected mice had previously been linked to the activation of Th2 responses stimulated by egg-derived antigens [38]. In accordance with data from our previous study, significantly higher antibody titres were occasionally detected in *Sm*+ D2 HMA compared to their WT counterparts (Fig. 3), a finding that might be attributable to the higher worm burdens recorded in the former group of rodents [23].

### Acute patent *S. mansoni* infection is associated with reduced bacterial diversity in the gut of D2 HMA mice

**Fig. 4:**
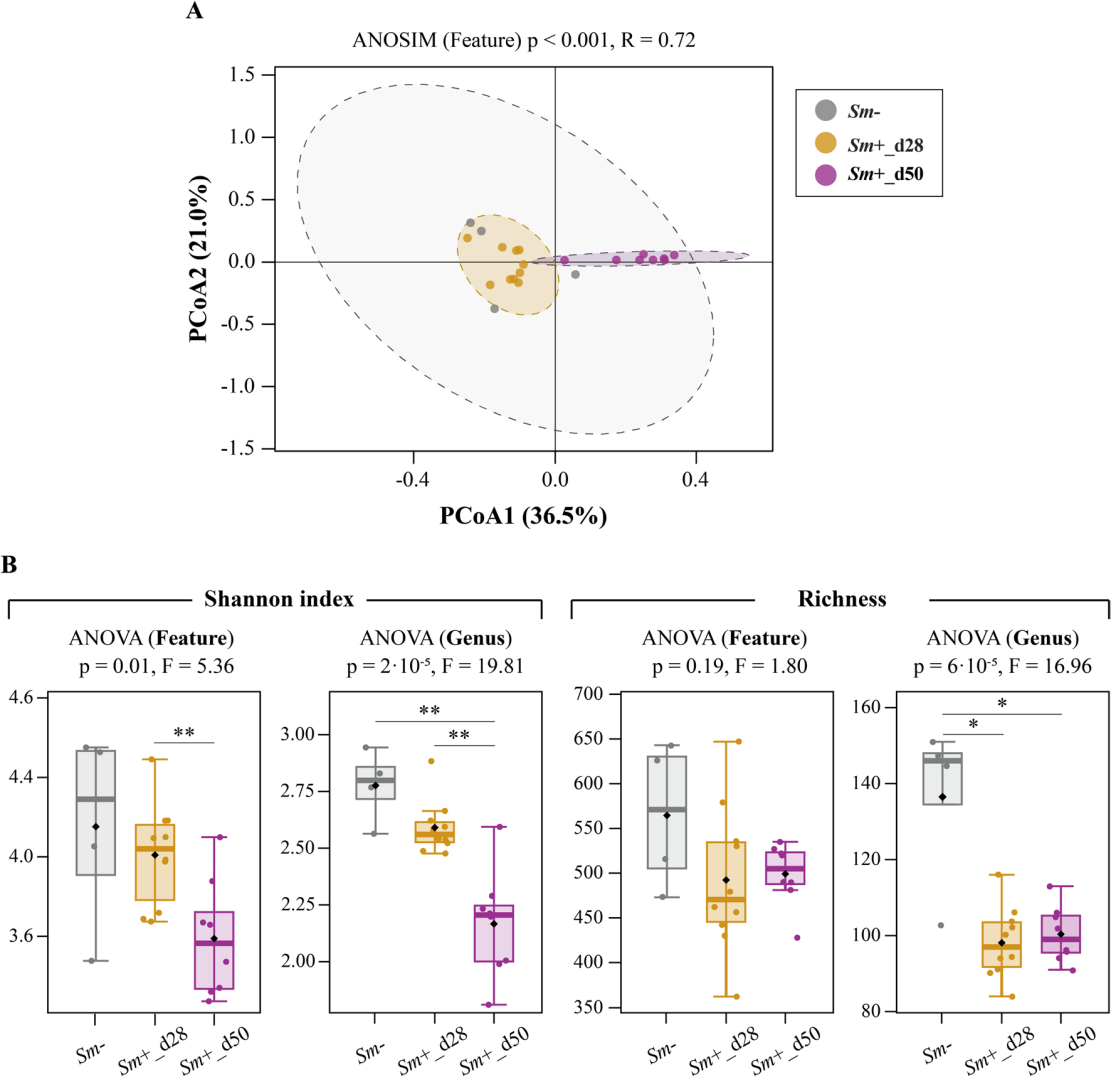
Infection with *Schistosoma mansoni* is associated with significant alterations of faecal microbial diversity in D2 human-microbiota-associated (HMA) mice. *S. mansoni* uninfected (*Sm−*; *n* = 4) and infected (*Sm*+) D2 HMA mice at 28 and 50 days post-infection [_d28 (*n* = 10) and _d50 (*n* = 8), respectively]. **A** Principal Coordinates Analysis (PCoA) based on Bray–Curtis distance. **B** Shannon index and richness at feature and genus level; error bars represent standard error of mean, and horizontal lines indicate significant differences between group pairs assessed by *t*-test: *p < 0.05; **p < 0.01

The impact of *S. mansoni* on the relative abundances and putative functions of microbial communities inhabiting the gut of experimentally infected rodents has been discussed elsewhere [23]. However, the alterations in microbiota composition that follow parasite colonisation are linked to the baseline structure and/or composition of the gut microbiota [23]. Therefore, to investigate similarities and differences between infection-associated changes in gut microbiota composition of distinct lines of HMA mice, we analysed the gut microbiota profiles of D2 HMA mice prior to and following the onset of egg laying. A total of 2,109,102 paired-end reads were generated from 22 faecal samples collected at different time points (i.e., 0, 28, and 50 dpi, see Supplementary Material 1) and subjected to further processing. Following read-merging, quality filtering and removal of chimeric sequences, a total of 1,096,635 high-quality sequences (per sample mean and standard deviation = 49,847 ± 13,916.8) were retained for feature table construction (Supplementary Material 3). These sequences were assigned to 2440 ASVs belonging to 97 bacterial genera and 10 phyla. Substantial differences in the relative abundances of several bacterial taxa were observed between the gut microbiota of *Sm−* and *Sm*+ mice at different post-infection time points (Supplementary Material 4). PCoA of gut bacterial profiles of *Sm*+ (28 and 50 dpi) and *Sm−* D2 HMA revealed separate clustering of *Sm*+_d50 samples (ANOSIM *p* < 0.001; R = 0.69) (Fig. 4A), thus reaffirming that the most substantial changes in gut microbiota composition occur during patent schistosomiasis [22, 39].

Significant differences in bacterial alpha diversity (Shannon index) were detected by ANOVA both at ASV and genus level (Fig. 4B). In particular, pairwise comparisons indicated that, at feature level, the rodent gut microbiota during patent *S. mansoni* infection was linked to a significantly reduced Shannon index compared to that of pre-patent mice (*p* = 0.007, *t* = 3.17) (Fig. 4B). At genus level, Shannon index was significantly decreased in *Sm*+_d50 compared to both *Sm−* (*p* = 0.007, *t* = 5.27) and *Sm*+_d28 (*p* = 0.001, *t* = 4.63), whilst no significant differences were detected between the latter two groups (*p* = 0.16, *t* = 1.61) (Fig. 4B). No significant infection-associated changes in microbial richness were detected amongst experimental groups at feature level (Fig. 4B). However, at genus level, bacterial richness was reduced in *Sm*+ (at 28 and 50 dpi) compared to *Sm−* (*Sm*+_d28 vs.* Sm−*: *p* = 0.04, *t* = 3.2; *Sm*+_d50 vs.* Sm−*: *p* = 0.04, *t* = 3.2; *Sm*+_d28 vs. *Sm*+_d50: *p* = 0.61, *t* = 0.53) (Fig. 4B). The observed reduction in microbial alpha diversity post-egg laying is consistent with our previous findings in both *Sm*+ WT and D7 HMA [23, 22], as well as in distinct mouse strains experimentally infected with *S. japonicum* [39].

Given that a high microbial alpha diversity is generally considered as an indicator of a ‘healthy’ gut [40, 41], findings from this and previous studies [22, 23, 39, 42, 43] suggest that schistosome infections exert a negative impact on the homeostasis of the gut microbiota of rodent hosts. In humans, microbial alpha diversity was significantly reduced in faeces from infected children from Côte d'Ivoire [44]*,* whilst the microbiota of Kenyan children showed no diversity changes linked to *S. mansoni* infection when compared to uninfected controls (reviewed by [45]). Variability across human populations and study designs may account for these discrepancies [46]. Nonetheless, it is likely that, both in mice and in humans, patent schistosomiasis negatively affects gut microbial homeostasis, possibly leading to microbiota dysbiosis with detrimental health consequences for the host [40, 41].

### *S. mansoni* acute patent infection is associated with expansion of hydrogen sulphide (H_2_S)-producing bacteria in the gut of D2 HMA mice

**Fig. 5:**
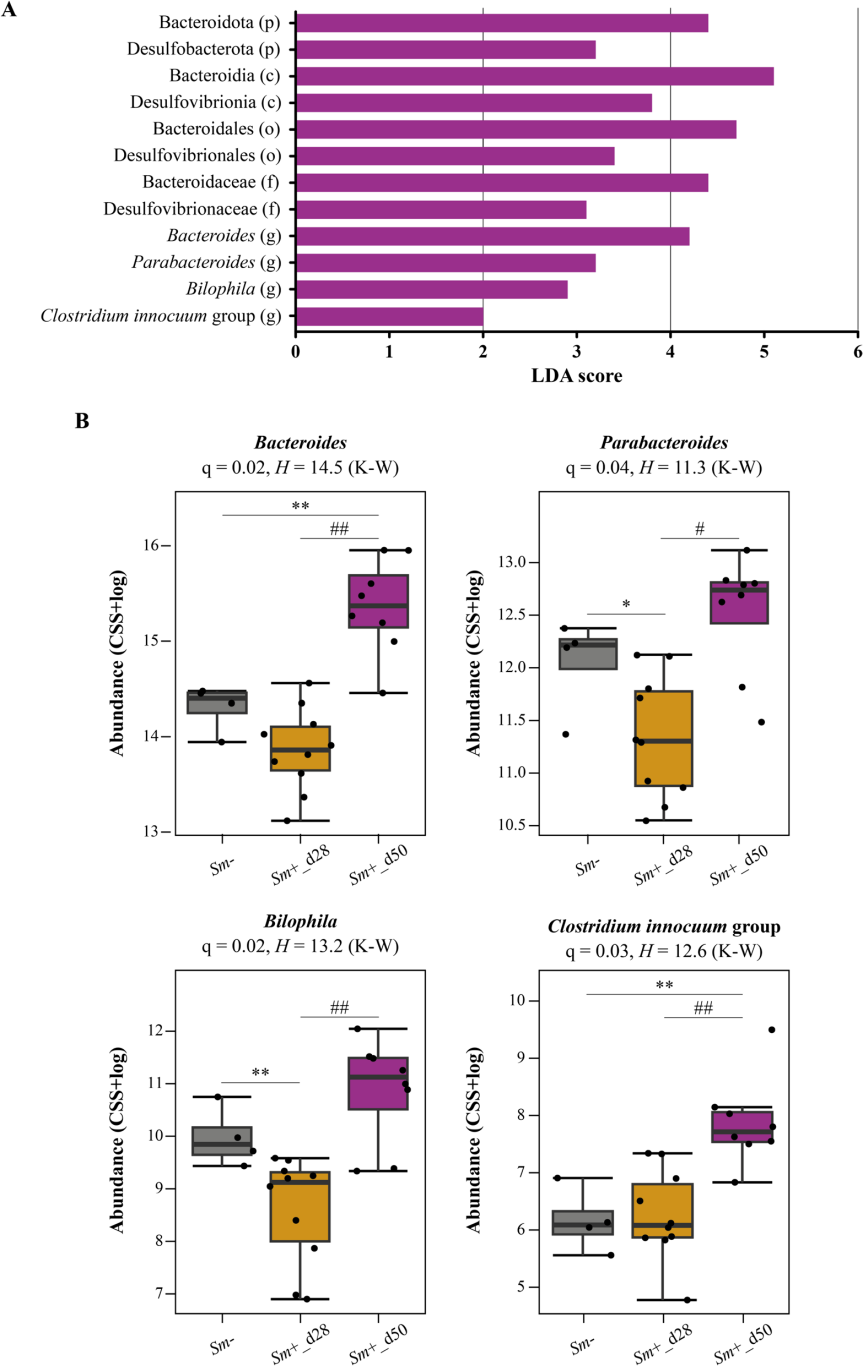
Acute patent *Schistosoma mansoni* infection is associated with expanded populations of selected groups of bacteria in faeces of D2 human microbiota-associated (HMA) mice. **A** Selected microbial taxa displaying significantly higher relative abundance in faecal samples from D2 HMA mice at 50 days post *S. mansoni* infection (*Sm*+_d50; *n* = 8), compared to samples collected from both uninfected mice (*Sm−*; *n* = 4) and at 28 days post infection (*Sm*+_d28; *n* = 10); results based on LEfSe (*q* < 0.05 and LDA score ≥ 2.0) and supported by Kruskal–Wallis (K-W) (*q* < 0.05) (see Supplementary Material 5). Taxonomic rank is indicated in brackets: (p) phylum, (c) class, (o) order, (f) family, and (g) genus. **B** Boxplots representing abundance of bacterial genera shown in **A**. Error bars represent standard error of mean and horizontal lines indicate significant differences between group pairs assessed by Wilcoxon rank-sum test (^#^*q* < 0.05; ^##^*q* < 0.01; *q > 0.05 and *p* < 0.05; **q > 0.05 and *p* < 0.01)

Infection-associated alterations in the relative abundances of individual bacterial taxa in the gut of D2 HMA mice were investigated using LEfSe, and verified by Wilcoxon rank-sum or Kruskal–Wallis test. Significant changes were detected at both 28 and 50 dpi compared to uninfected controls, as well as between pre-patent (28 dpi) and patent (50 dpi) infections (Supplementary Material 5). A marked expansion of bacteria belonging to the family Bacteroidaceae and genus *Bacteroides*, along with the family Tannerellaceae and genus *Parabacteroides* (all belonging to the order Bacteroidales) were observed in the gut of *Sm*+_d50 compared to both *Sm−* and *Sm*+_d28 (Fig. 5 and Supplementary Material 5). Alterations in populations of *Bacteroides* and *Parabacteroides* have been previously reported in the gut of laboratory rodents experimentally infected with *S. mansoni* [23, 24, 20, 42, 22, 43, 39].

Additionally, patent *S. mansoni* infection was linked to a significant expansion of *Bilophila* (Fig. 5)*,* a low-abundant genus of bacteria that includes a single accepted species (i.e., the intestinal pathobiont *B. wadsworthia*; [47, 48]), which commonly represents < 0.01% of the healthy human microbiota [47]. Overgrowth of *Bilophila* has been associated with inflammatory bowel conditions; for instance, a saturated fatty acid-rich diet induced significant expansions of *B. wadsworthia* that were associated with the onset of a pro-inflammatory Th1 response in the colonic mucosa and increased incidence of spontaneous colitis in genetically susceptible *Il10*^−/−^ mice [49]. The composition of the gut microbiota was shown to influence susceptibility to intestinal illness in the *Il10*^*−/−*^ mouse model of inflammatory bowel disease; remarkably, in these mice, *B. wadsworthia* was suggested to exacerbate intestinal pathology triggered by the mouse pathobiont, *Helicobacter hepaticus* [50]. Furthermore, oral administration of *B. wadsworthia* to conventional mice fed a fat-rich diet resulted in severe inflammation, intestinal barrier dysfunction and metabolic syndrome [51].

*Bilophila* belongs to the family Desulfovibrionaceae, a group of bile-resistant, low-abundant bacteria considered as major producers of hydrogen sulphide (H_2_S) in the gut lumen [52–54]. Likely linked to the expansion of *Bilophila*, overall proportions of Desulfovibrionaceae, and corresponding order, class, and phylum (Desulfovibrionales*,* Desulfovibrionia and Desulfobacterota, respectively) were also amongst the most significantly elevated taxa in the gut of *Sm*+_d50 compared to *Sm*+_d28 and *Sm−* (Fig. 5 and Supplementary Material 5). An excessive H_2_S production by the colonic microbiota has been linked to several gut conditions, such as inflammatory bowel disease and colorectal cancer, likely as a result of the known toxic effects of H_2_S on the intestinal epithelium that include reduction of mucosal integrity, inhibition of butyrate oxidation by colonocytes, and DNA damage [52, 53, 55]. Therefore, it is plausible that microbiota-derived H_2_S may aggravate infection-associated gut barrier dysfunction in D2 HMA mice (Figure 2), a hypothesis that requires thorough testing (e.g., via metagenomics and metabolomics analyses).

Acute patent infection was also associated with the expansion of other putative gut pathobionts, such as species of *Enterococcus* [56, 57] and of the *Clostridium innocuum* group [58] (Fig. 5 and Supplementary Material 5). Interestingly, both *B. wadsworthia* and *C. innocuum* were markedly overrepresented in the gut of HMA mice generated using faecal samples from Malawian children suffering from severe protein malnutrition (i.e., kwashiorkor), compared to mice transplanted with faeces of their corresponding healthy twins [59]. The aetiology of this disease, that affects children from the poorest regions of the world [60] where schistosomiasis is also endemic [61], remains unclear [60]. Nevertheless, the gut microbiome has been implicated as a potential contributing factor to kwashiorkor, with malnutrition affecting gut microbial function and thus further aggravating the disease. Moreover, it has been hypothesised that enteropathogenic infections may trigger microbiome disturbances that eventually lead to kwashiorkor [59]. Whether microbial imbalances linked to schistosomiasis may lead to increased risk of severe malnutrition in predisposed children from endemic areas is as yet unclear, and worthy of further investigation.

### Comparative analyses across mouse lines suggest a role for bile acids in modulating the infection-associated gut microbiota

In our previous studies of schistosome-infected D7 HMA, the qualitative and quantitative alterations in gut microbial profiles that followed parasite colonisation were strongly linked to baseline gut microbiota composition [23, 24]. Notwithstanding, the analysis of the metabolic capacity of the gut microbiome associated with *S. mansoni* infection in WT vs. D7 HMA mice revealed similarities between the two lines, thus supporting the hypothesis that parasite colonisation may induce consistent changes in gut microbial function, irrespective of the composition of the basal and/or infection-associated microbiota [24]. To further explore these findings and gather new insights into parasite-microbiota interactions in hepatointestinal schistosomiasis, we compared the faecal microbiota of *Sm−* and *Sm*+_d50 D2 HMA mice, with those reported previously in WT and D7 HMA mice at the same post-infection time point [23]. It is worth pointing out that, while the baseline gut microbial communities of these mouse lines are substantially different, these mice had been bred and housed in the same animal facilities, and experimentally infected using the same parasite strain, infection dose and procedures, thus minimising microbiome-unrelated variability across groups.

**Fig. 6:**
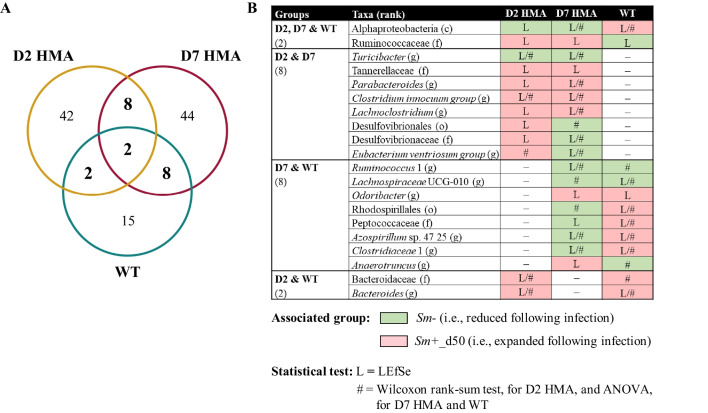
Differentially abundant faecal bacterial taxa in *Schistosoma mansoni* (*Sm*)-infected wildtype (WT) and D2 and D7 human microbiota-associated (HMA) mice. **A** Venn diagram representing relationships between faecal bacterial taxa significantly altered by infection in WT and D2 and D7 HMA mice, irrespective of the associated group [i.e., uninfected (*Sm−*) or at 50 days post infection (*Sm*+_d50)]. **B** Bacterial taxa significantly altered by infection in at least two of the three mouse lines, associated group, and statistical test(s) supporting significant differences in relative abundance. Taxonomic rank: class (c), order (o), family (f) and genus (g). Data for WT and D7 HMA were obtained from Cortés et al. [23]

Similar to our previous observations [23, 24], marked differences were observed between infection-associated changes in microbiota composition across mouse lines, albeit some consistencies could also be identified (e.g., *Parabacteroides* was significantly expanded in both *Sm*+ D2 and D7 HMA mice; Fig. 6). Moreover, network analysis yielded a positive correlation between the relative abundance of this genus of bacteria and *S. mansoni* infection in WT [23], and a significant expansion of *P. distasonis* was detected in the gut microbiota of these mice at 50 dpi [24]. In contrast, intestinal populations of *Bacteroides* were increased in the gut microbiota of *Sm*+ WT and D2 HMA (Fig. 6) and reduced in *Sm*+ D7 HMA [23, 24]. Significant expansions of faecal populations of *Bacteroides* and/or *Parabacteroides* have been repeatedly reported in schistosome-infected mice [20, 22–24, 39, 42, 43], alongside significant alterations of bacteria within the genus *Alistipes* [20, 23, 24, 39, 42, 43]. Intestinal populations of *Alistipes* were up- and down-regulated in *Sm*+ WT and D7 HMA mice, respectively, compared to their matched uninfected controls [23, 24]; conversely, no significant differences in *Alistipes* relative abundance were detected between the gut microbiota of *Sm−* vs. *Sm*+ D2 HMA mice.

*Bacteroides*, *Alistipes* and *Bilophila* – all expanded in the gut of *Sm*+ WT and/or D2 HMA at 50 dpi, but reduced (i.e., *Bacteroides* and *Alistipes*) or undetected (i.e., *Bilophila*) in D7 HMA (Fig. 6; [23, 24]) – are bile acid (BA)-resistant bacteria that thrive in the presence of elevated concentrations of BAs [62]. The gut microbiota composition and function and the BA pool are intimately interrelated [63–65]. Hence, the observed changes in populations of bile-resistant bacteria following *S. mansoni* infection – similar in WT and D2 HMA, and opposite to those seen in D7 HMA – point toward a likely role of the baseline microbiota/-me composition in the alterations of the BA profile during schistosomiasis that, in turn, might contribute to the observed dissimilarities between the gut microbial profiles of infected mice.

Schistosomiasis has been previously linked to alterations in BA synthesis and metabolism in rodent models [66–68]. Decreased levels of taurine, a cysteine-derived, sulphur-containing amino acid that conjugates with primary BAs in the liver [69], have been observed in urine from *S. mansoni* and *S. japonicum*-infected mice (at 49 and 35 dpi, respectively) [66, 67]. Accumulation of taurine was also detected in the liver of *S. japonicum*-infected mice at 35 dpi, suggesting that damaged liver tissue may be unable to form taurine-conjugated bile salts [67]. Notwithstanding, a recent study reported significantly elevated levels of total BAs, and of taurine-conjugated BAs in particular, in the liver of mice infected with *S. japonicum* [68]. Interestingly, supplementation of culture media with taurine-conjugated BAs increased schistosome oviposition in vitro, at least during the first week in culture [70]. Intestinal populations of *Bilophila* have been positively correlated with the production of taurine-conjugated BAs [49, 51, 64], whereas increased taurocholate may promote the growth of *Enterococcus faecalis* in an environment with limited concentrations of deoxycholate [56]. Of note, populations of both *Bilophila* and *Enterococcus* were significantly expanded in the faeces of *Sm*+_d50 D2 HMA (Fig. 5 and Supplementary Material 5).

Alterations of the BA pool in the liver of *S. japonicum*-infected mice were linked to significantly decreased expression of genes encoding several BA transporters (namely, *Bsep*, *Ntcp*, and *Ostβ*), as well as the BA-activated transcription factor FXR [68]. In *Fxr* knockout mice, *S. japonicum* infection led to further accumulation of BAs in the liver and exacerbated hepatic inflammation and injury when compared to WT; nevertheless, no significant differences in egg-induced liver granulomas and fibrosis were observed between WT and *Fxr* knockout mice [68]. In contrast, *Bsep*^−/−^ mice infected with *S. mansoni* displayed significantly reduced liver egg counts and granulomas compared to infected WT [71]. Moreover, adult worms recovered from *Bsep*^−/−^ mice displayed tegumental alterations, smaller male and female reproductive organs, and decreased parasite fecundity. Such a phenotype, indicative of impaired parasite development, might be linked to the altered BA profile and reduced blood pH that characterise these mice [71].

Altogether, this evidence supports the occurrence of close relationships between schistosome infection, the host BA profile, and both parasite development and disease pathogenesis. While the links between BAs and the gut microbiota are well known [63, 65], whether the baseline microbiota composition affects BA synthesis and/or metabolism in the liver of schistosome-infected mice remains unclear, as does the impact of the infection-associated microbiota on the BA pool. Interestingly, intraperitoneal inoculation of *S. japonicum* SEA to mice prior to induction of DSS-colitis reduced colonic inflammation and improved disease symptoms, an outcome that was accompanied by an increased production of selected BAs and their derivatives, amongst other alterations [72]. Furthermore, *Turicibacter*, a bacterial genus capable of processing BAs [73], was consistently decreased following infection in both D2 and D7 HMA mice (Fig. 6) and a negative correlation was previously observed between infection burdens and faecal populations of these bacteria in the latter mouse line [23]. Negative associations between *Schistosoma* spp. and intestinal populations of *Turicibacter* have been also reported in other studies [22, 39]. However, the BA-transforming capacity of *Turicibacter* varies amongst mice- and human-derived isolates [73] and, therefore, the consequences that decreased populations of these bacteria may exert on BA metabolism in each mouse line are difficult to predict with current data. However, since decreased populations of *Turicibacter* have been linked to colitis and reduced intestinal butyrate levels [74–76], future studies may focus on the potential contribution of such changes to intestinal inflammation in *Sm*+ mice.

### Faecal lipocalin and markers of alternatively activated macrophages display similar kinetics in *S. mansoni*-infected WT and HMA mice

Several inflammatory markers and cytokines were measured in faecal homogenates of schistosome-infected HMA at 28 and 50 dpi (D2 HMA) and 50 dpi (D7 HMA), as well as matched WT at corresponding time points. For this purpose, we processed any faecal samples that remained after 16S rRNA amplicon sequencing (see Supplementary Material 1), which led to a reduction of sample size that may have hindered the statistical power of some of our analyses (see below).

**Fig. 7:**
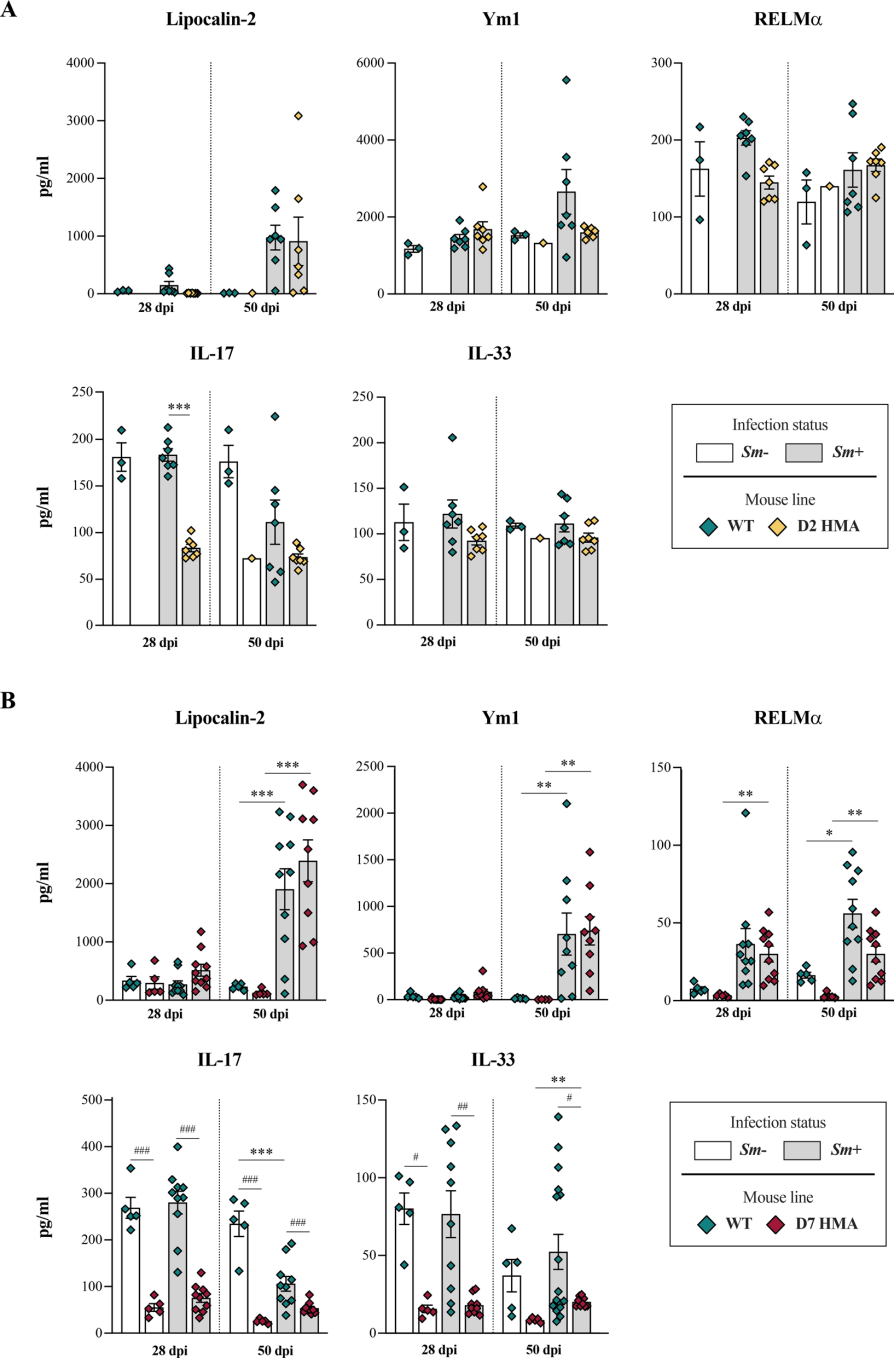
Baseline gut microbiota composition and infection with *Schistosoma mansoni* are associated with significant differences in levels of faecal cytokines and inflammatory markers. Concentrations of lipocalin-2, Ym1, RELMα, IL-17 and IL-33 in faeces of D2 (**A**) and D7 (**B**) human microbiota-associated (HMA) and matched wildtype (WT) mice at 28 and 50 days post infection (dpi) with *S. mansoni* (*Sm*+), compared to corresponding uninfected (*Sm*-) mice. *N* ranged between 0–5 *Sm−* and 7–10 *Sm*+ for each D2 and D7 HMA and WT at 28 dpi, and between 1–5 *Sm−* and 7–10 *Sm*+ for each D2 and D7 HMA and WT at 50 dpi (see Supplementary Material 1). Asterisks indicate significant differences between *Sm*+ and *Sm−* animals of the same line, and hash symbols between *Sm*+ animals of different mouse lines: */^#^*p* < 0.01, **/^##^*p* < 0.001, ***/^###^*p* < 0.001

Lipocalin-2, a non-invasive biomarker of intestinal inflammation [77], was significantly elevated in the faeces of *Sm*+_d50 D7 HMA and WT compared to their matched uninfected controls (Fig. 7). Increased levels of lipocalin-2 were also detected in faeces of *Sm*+ D2 HMA and matched WT mice at 50 dpi, compared to corresponding *Sm−*, albeit such differences did not reach statistical significance (Fig. 7). Lipocalin-2 is an innate immune peptide with anti-inflammatory and anti-oxidative properties involved in gut barrier protection and maintenance of gut microbiota homeostasis [78]. In *Il10*^−/−^ knockout mice, lipocalin-2 protects against spontaneous intestinal inflammation and tumorigenesis linked to pathogenic microbiota alterations [79]. The protective effects of lipocalin-2 are thought to be linked to an increased phagocytic capacity of macrophages and bacterial clearance [80]. Hence, increased levels of this inflammatory marker following the onset of egg laying may assist the restoration of gut barrier integrity against egg-mediated tissue injury and, potentially, protect against any pathogenic effects caused by gut resident bacteria.

Ym1 was significantly elevated in faeces of *Sm*+_d50 D7 HMA compared to uninfected mice; a similar trend was observed in matched WT samples, albeit no statistical significance was recorded in the latter (Fig. 7). In addition, RELMα was increased in faeces from *Sm*+ WT and D7 HMA at both 28 dpi and 50 dpi in comparison to *Sm−* at corresponding time points (Fig. 7). Increased production of these markers likely reflects a Th2-mediated alternative activation of macrophages in response to infection, that has been shown to protect mice from severe egg-induced intestinal and liver pathology during acute schistosomiasis [81]. In C57BL/6 mice, Th2-dominated responses against SEA also exert a protective role against severe pathology induced by Th17 cells [82]. Accordingly, levels of IL-17 were decreased in faeces from *Sm*+ WT mice at 50 dpi (Fig. 7). However, strikingly, faecal levels of IL-17 were higher in WT mice in comparison to either infected and uninfected D2 and D7 HMA at both 28 and 50 dpi (Fig. 7). Similarly, faecal IL-33 was elevated in *Sm−* and *Sm*+ WT mice compared to corresponding D7 HMA samples, both at 28 and 50 dpi, while no differences in faecal IL-33 levels were observed between WT and D2 HMA (Fig. 7).

Increased levels of faecal lipocalin and/or markers of alternatively activated macrophages, as well as of serum antibodies in response to *S. mansoni* infection, suggest that, like WT mice, HMA mice mount innate and adaptive responses against this parasite. Nevertheless, reduced levels of faecal IL-17 and IL-33 in naïve HMA mice compared to WT raise the question of whether microbiota-dependent differences in baseline immunity between lines may be responsible for the higher worm burdens observed in microbiota-humanised rodents. Indeed, the mammalian gut microbiome is essential for the maturation and functioning of the immune system [83, 84] and HMA mice are known to display altered immune responses and susceptibility to intestinal bacterial infections [85–87]. To further investigate these aspects, it is important to determine the exact phase of intra-mammalian development in which schistosomes are eliminated in comparatively higher numbers in WT vs. HMA mice. In WT rodents experimentally infected with *S. mansoni,* parasite elimination has been reported to occur mainly during the lung migration stage [88]; hence, our data suggest that survival of lung-migrating schistosomula may be greater in HMA compared to WT mice, thus leading to the establishment of a larger number of worm pairs in the liver at ~ 28 dpi [89] (Figure 1). Nonetheless, given the substantial contribution of the skin microbiome to epidermal barrier formation and differentiation [90], it cannot be ruled out that the higher worm burdens observed in HMA compared to WT mice may (also) result from greater efficiency in skin penetration by the invading cercariae. Further analyses of skin and pulmonary immune responses to *S. mansoni* in WT vs. HMA mice are likely to provide entirely novel insights into the impact of the host microbiome on susceptibility to schistosomiasis. Additionally, HMA rodents recolonized with WT-derived faecal microbiota prior to infection may assist the evaluation of the ability of the latter to ‘restore’ the host capacity to control infection, as well as the identification of key bacterial taxa linked to the higher parasite burdens observed in HMA.

## Conclusion

Supported by previous studies, we show that, irrespective of baseline gut microbiota composition, migration of schistosome eggs through the intestinal wall, and/or their accumulation in the liver, exert a major impact on gut microbial homeostasis, generally leading to a decrease in bacterial alpha diversity that might worsen the health status of infected animals. Furthermore, although this study focuses on changes in relative abundances of bacterial taxa associated with schistosome infection, our results provide further support to the hypothesis that the baseline gut microbiota may influence the host ability to control parasite burdens, at least in its early phases [23], and represent a risk factor for comorbidities of multifactorial nature, such as severe malnutrition and environmental enteric dysfunction, both of which are common in low-income countries where schistosomiasis is endemic [23, 60, 91–93]. The specific traits of the gut microbiota and underlying mechanisms that influence susceptibility to schistosomiasis and microbiome-related comorbidities remain to be investigated. In the future, in-depth time-course studies of the immune response against schistosome infection in HMA mice, including local responses occurring in the skin and lungs (sites of parasite penetration and migration, respectively), will be necessary to clarify these aspects. For instance, correlation analyses between bacterial taxa abundances and levels of immune mediators produced immediately post-cercarial invasion and during the lung migration phase (i.e., from day 0 to day 21 post-infection [94, 95]) may assist the identification of key links between host baseline microbiota composition and immunity to infection.

Moreover, causal relationships that may occur between gut microbial imbalances in individuals from endemic areas, particularly children [44, 60, 92], and susceptibility to disease deserve to be thoroughly investigated. This hypothesis may be explored in large-scale, longitudinal studies (similar to the MAL-ED study [96]) involving newborns from a range of schistosomiasis-endemic geographical areas. In such studies, history of infection and deworming, as well as gut microbiome composition, could be closely monitored throughout the first years of life alongside other variables that may influence gut microbial makeup (e.g., unrelated infections and illnesses, antibiotic use, and/or dietary practices). Establishing causal relationships will nonetheless require rigorous follow-up testing of the effect(s) of bacterial species/strains identified in such epidemiological studies using laboratory models of infection (e.g., via supplementation of WT and HMA mice with selected bacteria prior to infection and subsequent comparison of parasite burdens and immune responses with non-supplemented mice). Investigating infection-associated changes in gut microbiota composition and immunity in rodents re-colonised with the microbiota of several human hosts, particularly from schistosomiasis-endemic areas, may also contribute to this goal.

Comparisons across WT, D2 and D7 HMA mice indicated that infection-induced alterations of the gut microbiota composition are highly influenced by the baseline flora, with only a few consistencies identified amongst mouse lines. In spite of such variability, our observations point towards BAs as potentially key modulators of gut microbial communities during schistosomiasis. Furthermore, the basal microbiota composition has emerged as a likely important factor influencing BA profiles in schistosome-infected mice. These findings, together with previous evidence of the impact of infection on BA homeostasis and the consequences of altered BA pools for parasite biology and pathogenesis (e.g., [71, 68]), support the need for studies aimed to characterise BA profiles in WT and HMA mice, and to explore mechanisms of host-parasite-microbiota interactions through the gut-liver axis.

### Supplementary Information

Supplementary Material 1. Number of biological samples (*n*), grouped according to mouse line and (pre- and post-patent) infection status, as well as to sample origin (i.e., present study vs. Cortés et al. [23]) processed for parasitological analyses, bacterial 16S rRNA amplicon sequencing, serological and faecal inflammatory marker assays.

Supplementary Material 2. Serum/faecal and antibody dilutions used for ELISA assays. SEA: soluble egg antigen; SWAP: soluble worm antigen preparation.

Supplementary Material 3. Sequencing results per sample (**A**) and experimental group (**B**). Number of reads prior to (i.e., 'raw') and following (i.e., 'combined') paired-ends (PE) merge, quality filtering (i.e., 'qualified') and chimeras' removal (i.e., 'Nochime'), percentage of bases with quality scores ≥ Q20 and Q30, and GC content (%).

Supplementary Material 4. *Schistosoma mansoni* infection is associated with substantial alterations of faecal microbial profiles of D2 human microbiota-associated (D2 HMA) mice. Relative abundance of the most abundant bacterial phyla (**A**) and genera (**B**) detected in faecal samples of *S. mansoni* uninfected (*Sm*−; *n* = 4) and infected (*Sm*+) D2 HMA mice at 28 and 50 days post cercarial exposure [_d28 (*n* = 10) and _d50 (*n* = 8), respectively].

Supplementary Material 5. Faecal bacterial taxa altered following *Schistosoma mansoni* (*Sm*) infection in faecal samples of D2 human microbiota-associated (D2 HMA) mice. Results of LEfSe and Kluskal-Wallis/Wilcoxon rank-sum test applied to the identification of differentially abundant bacteria between the faecal microbial communities of uninfected (*Sm*−; *n* = 4) and infected (*Sm*+) mice at 28 and 50 days post cercarial exposure [_d28 (*n* = 10) and _d50 (*n* = 8), respectively]. Taxa displaying *p*-value < 0.05 and LDA score ≥ 2 by LEfSe, and/or *p*-value < 0.05 by Kluskal-Wallis/Wilcoxon rank-sum test are included. **A** Summary of differentially abundant bacterial taxa as determined by LEfSe. **B-E** Statistically significant differences determined by LEfSe and/or Kluskal-Wallis/Wilcoxon rank-sum following comparative analyses of: **B** the three experimental groups (*Sm*−, *Sm*+_d28 and *Sm*+_d50); **C** microbiota profiles of *Sm*- mice and at 28 days post infection (*Sm*+_d28); **D** microbiota profiles of *Sm*- mice and at 50 days post infection (*Sm*+_d50); and **E** microbiota profiles of *Sm*+_d28 and *Sm*+_d50.
