# Supplementary figures and images for: Gut microbiota and immune profiling of microbiota-humanised versus wildtype mouse models of hepatointestinal schistosomiasis
Source: Anim Microbiome. 2024 Jun 25;6:36. doi: 10.1186/s42523-024-00318-3 (PMC11201864; doi:10.1186/s42523-024-00318-3)

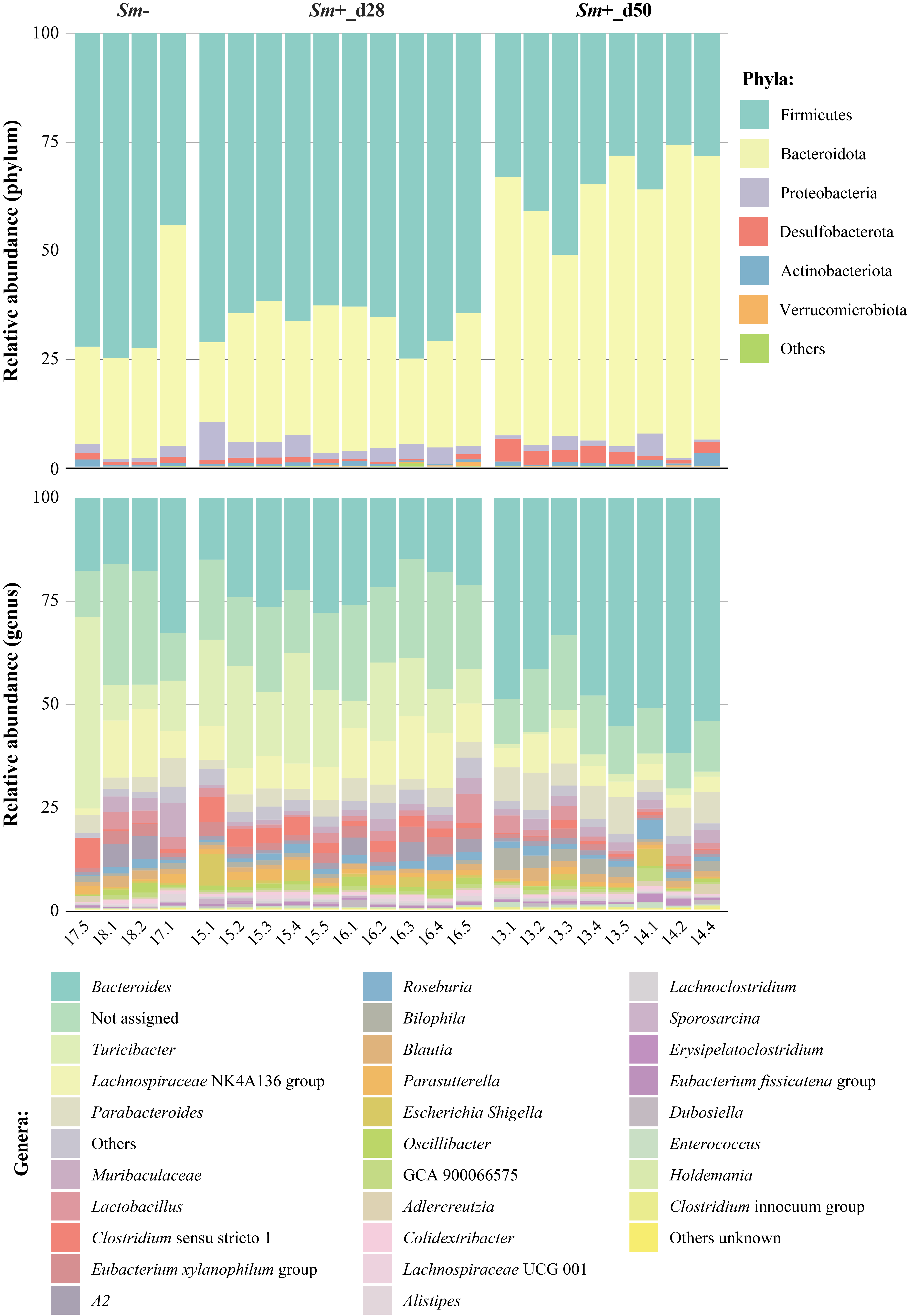

Supplement: Supplementary file 4 — Supplementary Material 4. Schistosoma mansoni infection is associated with substantial alterations of faecal microbial profiles of D2 human microbiota-associated (D2 HMA) mice. Relative abundance of the most abundant bacterial phyla (A) and genera (B) detected in faecal samples of S. mansoni uninfected (Sm−; n = 4) and infected (Sm+) D2 HMA mice at 28 and 50 days post cercarial exposure [_d28 (n = 10) and _d50 (n = 8), respectively]. [file 42523_2024_318_MOESM4_ESM.png]
